# Ten Priorities for Research Addressing the Intersections of Brain Injury, Mental Health and Addictions: A Stakeholder‐Driven Priority‐Setting Study

**DOI:** 10.1111/hex.14136

**Published:** 2024-07-11

**Authors:** Cole J. Kennedy, Erica Woodin, Julia Schmidt, Janelle Breese Biagioni, Mauricio A. Garcia‐Barrera

**Affiliations:** ^1^ Department of Psychology University of Victoria Victoria Canada; ^2^ Institute on Aging & Lifelong Health University of Victoria Victoria Canada; ^3^ BC Consensus on Brain Injury, Mental Health, and Addiction Victoria British Columbia Canada; ^4^ Canadian Institute for Substance Use Research University of Victoria Victoria Canada; ^5^ Department of Occupational Science and Occupational Therapy, Faculty of Medicine University of British Columbia Vancouver Canada; ^6^ Rehabilitation Research Program Centre for Aging SMART, Vancouver Coastal Health Research Institute Vancouver Canada; ^7^ CGB Centre for Traumatic Life Losses Victoria Canada

**Keywords:** acquired brain injury, addiction, mental health, priority‐setting, stakeholder engagement, substance use

## Abstract

**Objectives:**

The purpose of this study was to engage key stakeholders in a health research priority‐setting process to identify, prioritize and produce a community‐driven list of research questions addressing intersectional issues on mental health and addictions (MHA) in acquired brain injury (ABI).

**Methods:**

A multiphasic health research priority‐setting process was co‐designed and executed with community‐based stakeholders, including researchers, health professionals, clinicians, service providers, representatives from brain injury associations, policy makers and people with lived experience of ABI and MHA, including patients and their family members. Stakeholders' ideas led to the generation of research questions, which were prioritized at a 1‐day workshop.

**Results:**

Fifty‐nine stakeholders participated in the priority‐setting activity during the workshop, which resulted in a rank‐ordered list of the top 10 questions for research addressing the intersections of ABI and MHA. Questions identified touched on several pressing issues (e.g., opioid crisis, homelessness), encompassed multiple subtypes of ABI (e.g., hypoxic‐ischaemic, mild traumatic), and involved different domains (e.g., identification, intervention) of health research.

**Conclusions:**

This community‐driven health research priority‐setting study identified and prioritized research questions addressing the intersections of ABI and MHA. Researchers and funding agencies should use this list to inform their agendas and address stakeholders' most urgent needs, fostering meaningful improvements to clinical services.

**Patient or Public Contribution:**

An 11‐person working group comprised of people with lived experience, service providers, researchers, healthcare professionals and other key stakeholders collaboratively developed and informed the scope, design, methodology and interpretation of this study. Over 50 community‐based stakeholders contributed to the research priority‐setting activity. One co‐author is a person with lived experience.

## 1 Introduction

Acquired brain injury (ABI) is the umbrella term referring to a wide array of neurological insults and injuries, including traumatic brain injury (TBI), cerebrovascular incidents (stroke and other ischaemic events), hydrocephalus, cerebral infections, tumours, hypoxia/anoxia, etc. It is a highly prevalent condition, affecting well over 80 million people each year and costing the global economy upwards of 900 billion (US) dollars annually [1, 2, 3, 4, 5]. Mental health and addiction (MHA) problems, such as anxiety, depression and substance use disorders are common following ABI [6, 7, 8, 9]. For example, approximately one‐third of stroke survivors experience post‐stroke depression [10], including feelings of apathy, irritability and sleep disturbance, which are known to impede rehabilitation and negatively affect survivors' quality of life [11]. In general, the risk of developing any MHA disorder is increased twofold following ABI [12] and is associated with significant morbidity and mortality [13, 14].

Changes in psychosocial well‐being, physical mobility, autonomy, and underlying neural circuitry all contribute to how a person responds after ABI [6, 9, 15, 16]. One such post‐injury coping response is the use of illicit substances or misuse of alcohol or prescription medication [17, 18, 19], which can lead to problematic substance use in the form of addiction [17, 19, 20]. Therefore, people who experience ABI are at an elevated risk for developing substance dependence after their injuries [17, 21, 22], even for those who sustain their injuries in childhood [22]. Moreover, substance intoxication is also the leading contributor to accident‐related brain injury [9, 23], making it not only an outcome but also a predictor for ABI [6, 9, 23]. Despite being the third most common psychiatric outcome, far less research exists on substance use and addiction‐related disorders in ABI populations in comparison to depression, anxiety and other behavioural problems.

In general, epidemiological data on MHA problems after ABI are hindered by several issues. First, identifying MHA problems in ABI populations can be challenging as both issues share many common symptoms (e.g., headache, dizziness, fatigue, and irritability), making it difficult for healthcare providers to disentangle overlapping symptomatology [9, 24]. Second, some populations known to have high rates of ABI are also disproportionately affected by MHA, such as people who are experiencing homelessness [25, 26, 27, 28] or who experience intimate partner violence [29, 30, 31], confounding the recognition and cause‐and‐effect issues. In Canada, ABI is not tracked by the Chronic Disease Surveillance System, hence there is a lack of longitudinal data on multimorbidity and long‐term outcomes [32].

Understanding the intricate links between MHA and ABI remains a complex challenge. Indeed, bettering the lives of the populations they study should be researchers' foremost aspiration, but stakeholder guidance is needed to produce meaningful research with practical utility for knowledge‐end users. Research priority‐setting refers to a range of activities that involve identifying, prioritizing and achieving consensus on the research areas or questions of importance to stakeholders. These activities aim to ascertain what knowledge is most valued by patients, practitioners or the public as they become experts in their own healthcare experiences [33]. Several large organizations have established approaches with this purpose in mind, such as the James Lind Alliance [34] or the Global Evidence Mapping (GEM) Initiative [35, 36]. Research groups have taken differing approaches in their grounding frameworks, methodological designs and levels of stakeholder engagement, often devising novel methods best suited to their own objectives and needs [33, 37, 38, 39]. Leaders in the field suggest that frameworks, stakeholder engagement, approaches to prioritization and desired outcomes will depend largely on the topics of interest and the specific stakeholder groups involved [40, 41]. While there is no gold standard approach to research priority‐setting [42], all exist under the assumption that patients and the public should have a say in determining research priorities and decision‐making processes.

In the field of ABI, the limited number of health research priority‐setting studies have involved different stakeholder groups for different purposes. For example, the GEM initiative [35, 36] sought to understand patients' and clinicians' priorities for TBI and spinal cord injury rehabilitation to guide their series of knowledge syntheses. Similarly, Poulin et al. [43] surveyed ABI clinicians only to better understand their priorities towards ABI rehabilitation. Varying methods have also been used to set priorities. Solbakken et al. [39] developed and executed their own approach inspired by the James Lind Alliance [34], and involved a broad group of stakeholders through a series of surveys to identify a top‐10 list of research needs regarding transitional care for patients with acute stroke. Using the World Café method, Nalder and colleagues [44] hosted a workshop to collaboratively set priorities among different stakeholder groups for optimizing long‐term community integration after TBI. Although providing key fundamental data, these studies did not use a holistic approach with broad stakeholder engagement, and the perspectives of people with lived experience were underrepresented.

Many reviews have concluded that more research is needed to better understand the complex interconnections between ABI and MHA [9, 10, 11, 12, 22, 24], and an environmental scan of the evidence base shows that this need is more than justified [45]. However, exactly ‘what’ questions need answering is less clear. Health research priority‐setting processes have been applied to various forms of disease and disability, including MHA [46, 47, 48] and ABI [35, 36, 39, 43, 44] related issues, yet, until now, no priority‐setting study has been conducted to understand stakeholders' priorities for future research addressing the intersections of ABI and MHA. The lack of collaborative priorities set by representatives of diverse stakeholder groups, especially people with lived experience, represents a significant evidence gap. Meaningful inclusion of people with lived experience in health research priority‐setting is crucial for formulating purposeful and equitable research with high community and clinical impact. Therefore, the purpose of this study was to engage key stakeholder groups, including multidisciplinary researchers, clinicians, health professionals, service providers, brain injury association representatives, policy makers, health administrators, brain injury survivors and their caregivers or family members, in a health research priority‐setting process to identify, rank and produce a community‐driven list of priorities to guide future research addressing the intersections of ABI and MHA.

## 2 Methods

### 2.1 Study Design

The Council on Health Research for Development (COHRED) framework for priority‐setting in research for health was used to inform the design of the current study [49]. In addition to the COHRED framework, Viergever et al.'s checklist for good practice in health research priority‐setting [50] and Tong et al.'s REPRISE guidelines [40] were applied and followed. Additionally, several techniques and procedures were adapted from the GEM Initiative's method for research priority‐setting in the fields of TBI and spinal cord injury [35, 36]. All recruitment processes followed the best practices in equity, diversity and inclusion in research as recommended by the New Frontiers in Research Fund and the Canadian Research Coordinating Committee. Approval for this study was obtained from the University of Victoria (#22‐0614) and the University of British Columbia (#H22‐03403) Human Research Ethics Boards.

### 2.2 Working Group

The working group was established to both contribute to the goals of the priority‐setting process and to oversee its development, delivery and dissemination. The 11‐person group included three people with lived experience (two survivors of brain injury and one family member), three service providers, six scientist‐practitioners (i.e., psychologist, neuropsychologist, neuropsychologist trainee, two occupational therapists, and a registered clinical counsellor) and two other community‐based stakeholders (several individuals identified as more than one type of stakeholder). This composition was intentional to ensure equitable representation from different stakeholder groups. Importantly, knowledge from lived experiences (i.e., *emic* perspectives) and knowledge from more traditional forms of expertise through research and academia (i.e., *etic* perspectives) were valued equally. Strategies were implemented to reduce the potential influence of power imbalances (e.g., each meeting was led by a person with lived experience) and by fostering an environment of mutual respect, understanding and compassion. The group met monthly (or more frequently, as needed) over 14 months and was responsible for: (1) determining scope, (2) informing study design, (3) contributing to question generation activities, (4) reviewing and consulting on revised questions for prioritization and (5) confirming the final list of research priorities. Members who identified as people with lived experience (i.e., survivors and family members) were compensated for their time according to BC Centre for Disease Control (CDC) Peer Payment Standards [51].

### 2.3 Participants

Purposeful sampling was used. Participants were stakeholders of diverse backgrounds and experiences. In this study, ‘stakeholders’ included researchers of varying expertise, multidisciplinary health professionals, service providers, leaders and representatives from brain injury organizations, survivors of brain injury, policy makers, including politicians and health administration government officials, and family members of those affected by concurrent ABI and MHA related complications. We sought to include a broad cross‐section of stakeholders as each group possesses unique insights that must be considered. Participants were invited to attend ‘Consensus on Brain Injury Day’, a 1‐day workshop organized by our team as part of the BC Consensus on Brain Injury, Mental Health and Addiction research project (hereafter referred to as the BC Consensus on Brain Injury). Invitations were made with a particular focus on the inclusion of equity‐deserving groups, such as persons with disabilities, members of Indigenous groups and members of the LGBTQ2IA+ community, as these groups have been largely underrepresented in ABI research [52, 53]. There were no explicit exclusion criteria, other than no relation to our topic(s) of interest.

### 2.4 Priority‐Setting Procedures

The following section describes the multiphasic, multistep, multi‐informant and multiformat priority‐setting processes used in this study. As previously mentioned, our procedures were adapted from and informed by several pre‐established methods. In line with previous priority‐setting research [33, 37, 39, 41, 42, 44], a range of components of different methods were incorporated and several elements were uniquely devised to create an approach that was best suited to fit our guiding principles, study objectives, topic of interest and desired level of stakeholder engagement.

#### 2.4.1 Step 1: Expert Consultation

To inform the beginning stages of this study, it was important to first get a grasp on the intersections of ABI and MHA from experts in the field through open and ongoing dialogue. In congruence with the community‐engaged approach to this study, ‘experts’ were researchers, service providers, clinicians and people with lived experience, many of whom were members of the working group. For our purposes, ‘consultation’ involved meetings, informal conversations and review of previous works within our network. In addition, a University of Victoria librarian with experience in these fields and expertise in literature searching was consulted. While intangible and diversified, expert consultation was a critical step in determining our scope (e.g., objectives and goals), parameters (e.g., recruitment and compensation planning) and methodology (e.g., prioritization activity design and data analyses). Presented here as the first step, expert consultation carried throughout the entire course of this study and was fundamental to its success.

#### 2.4.2 Step 2: Preliminary Literature Review

The purpose of the literature review was to inform the development of a preliminary list of broad research topics described as priority areas in the existing literature. Common search phrases were identified from key articles and relevant subject headings and terms were mined from and adapted as required across information sources. Two major databases, MEDLINE (Ovid) and PsycINFO (EBSCOhost) were searched on 16 August, 2022 for relevant articles (see Supporting Information S1: Table 1 for search terms). To limit publication bias and identify ‘non‐elitist’ information, we also conducted an informal search of grey literature (e.g., unpublished research, editorials, government reports, policy literature, etc.), predominately from North American sources. Manual (hand) searching was also conducted. Expert consultation supported these processes, including the identification of key articles and grey literature sources. After reviewing the literature, relevant ‘calls to action’, knowledge gaps and recommendations for future research were extracted.

#### 2.4.3 Step 3: Topic Generation

The purpose of the topic generation phase was to formulate a list of priority research topics combining ideas from the literature with stakeholders' priorities. Raw text excerpts of identified knowledge gaps and calls to action were synthesized and collated in NVivo 12 [54] by the lead author to begin forming topics. In contrast to questions, ‘topics’ are broad ideas addressing research. For our purposes, topics served as somewhat discrete areas for stakeholders to focus their priorities around. The initial list of topics was distributed to the working group, who were first tasked with reviewing the list; revising, critiquing or rephrasing based on their knowledge and personal experiences. They were then instructed to add their own priorities or eliminate ones from the list, at their discretion. Revisions and feedback from this exercise were compiled in preparation for the next step.

#### 2.4.4 Step 4: Question Generation

The purpose of the question generation phase was to gather and formulate research questions based on stakeholders' needs, opinions, and of course, priorities. To facilitate this process, the list of research topics created through the previous step was circulated to the working group along with instructions and a question generation worksheet to help structure the task. To generate poignant research questions, stakeholders were encouraged to use the PICO (‘Population’, ‘Intervention’, ‘Comparator’, ‘Outcome’) format. The PICO format allows for structured research questions and has been used in research priority‐setting activities with stakeholders [35, 36, 55]. To help stakeholders formulate their questions, a ‘PICO Guide’ was also created and circulated to the working group. Use of the PICO format was encouraged, but not mandatory, and assistance from research personnel was made available.

#### 2.4.5 Step 5: Question Development

The purpose of the question development phase was to transform responses into a list of ‘answerable’ research questions that best reflect stakeholders' collective priorities. Responses from the question generation phase led to a large volume of unstructured data, with numerous individual written responses in the form of questions, ideas and statements all varying in their language, format and areas of emphasis. To convert heterogenous and unstructured responses expressing broad stakeholder perspectives into answerable questions, the GEM method of transforming PICO fragments was adapted and applied [35, 36]. By adopting a systematic approach to question development, we ensured that the integrity of stakeholders' ideas was maintained across the synthesis process. Following and expanding upon the GEM method, we used a nine‐step systematic process (see Table 1) to transform data into structured research questions.

**Table 1:** Steps for transforming question fragments during question development.

Step	Description of tasks involved
1	Entering the data into an electronic database (Microsoft Excel).
2	Coding PICO fragments and ideas using codes from the International Classification of Functioning, Disability and Health (ICF) and the International Classification of Diseases (ICD) in NVivo 12.
3	Organizing the coded data to identify the range and frequency of all codes. Examining for overlap among fragments, reducing redundancies.
4	Constructing initial questions from coded PICO fragments.
5	Coding each question as one of the following: diagnosis, prognosis, interventions or service delivery and organization.
6	Intercoder consultation to reduce the number of questions and ensure equal representation across the four categories (above).
7	Refining questions, ensuring consistent terminology and using layperson language whenever possible.
8	Consulting with the working group to further refine the questions.
9	Producing the final list of questions for prioritization.

*Note:* Adapted from the GEM method of transforming PICO fragments [35, 36].

#### 2.4.6 Step 6: Question Prioritization

Participants attended the BC Consensus on Brain Injury workshop on 14 October 2022, in British Columbia, Canada. The purpose of the question prioritization activity was to understand stakeholders' priority assessment of the identified research questions; that is, what questions they would like to see answered first. The questions were mounted to a web‐based survey in preparation for the workshop. The survey contained two sections from which data was collected. The first section, ‘ranking’, required participants to rank‐order the questions by priority. The second section, ‘rating’, required participants to rate each question based on its level of (1) *clinical importance* (how important the question is for improving health/healthcare), (2) *novelty* (how much the question represents an emerging field of interest or attention) and (3) *controversy* (the level of disagreement regarding opinion and/or practice for the question) on a sliding Likert scale of 1–5 (*not at all* to *high*). This rating procedure was adapted from the GEM method for prioritization [35, 36, 55]. Question prioritization was facilitated during the second half of the workshop and took approximately 20 min to complete. Participants were provided with instructions before starting the activity and facilitators were present to support them as needed.

### 2.5 Data Analysis

Descriptives were computed for participant demographic characteristics as well as for ranking and rating responses. Participants who completed the ranking section of the prioritization survey but did not progress to the rating section were dropped from all statistical analyses, as both rankings and ratings were required for our analyses. Additionally, cases with > 50% missing data were removed and multiple imputation was used to fill any remaining missing values [56]. This method provides more validity than other approaches to addressing missing data by using all available data to result in unbiased estimates [56, 57].

Lastly, we explored potential differences in priority evaluations between participants who identified as people with lived experience (i.e., survivors of brain injury and family members) and participants who did not (i.e., service providers, researchers, healthcare professionals, public safety workers, and government representatives) by calculating Kendall's coefficient of concordance (*W*) and point‐biserial (*r*_pb_) correlations and for all rankings and ratings, respectively.

The *Question Priority Composite* (*QPC*) equation was created by the research team and driven by our imperatives for comprehensively understanding stakeholders' priorities. To effectively do so, we felt that having multiple sources of input (i.e., rankings *and* ratings) was important for determining the level of priority for each question. Therefore, for each participant (*n*), individual question (*k*) ranking and rating scores were combined to formulate the *QPC* score. Rather than relying on ranking or rating scores alone, we believe that incorporating both variables produces a more robust score that considers the nuances of participants' priority evaluation of each research question. With these foundational goals in mind, the *QPC* formula was created and is as follows:

$${\mathrm{QPC}}_{n,k}=x_{\mathrm{rank}}+x_{\mathrm{rate}}$$

The first component of the *QPC* equation, $x_{\mathrm{rank}}$, represents ranking scores. Within the $x_{\mathrm{rank}}$ equation, ‘rank’ signifies the position in which participants ranked each question (i.e., 1 = highest priority, 2 = second highest priority, etc.), *q* is the total number of research questions, and *q* + 1 (numerator) serves to reverse score rankings, such that higher scores reflect higher priority. The denominator (*q*) reflects the highest possible ranking value and serves to balance the equation. $x_{\mathrm{rank}}$ is computed as follows:

$$x_{\mathrm{rank}}=\frac{\left\{\left(q+1\right)-\mathrm{rank})\right\}}{q}$$

The second component of the *QPC* equation, $x_{\mathrm{rate}}$, represents rating scores. Within the $x_{\mathrm{rate}}$ equation, clinical importance (*CI*), novelty (*nov*) and controversy (*cont*) are weighted. The decision to weigh these was guided by the GEM method for question prioritization, whereby they classified ‘high priority questions’ as those which participants rated clinical importance as ‘high’ AND novelty OR controversy as ‘high’ or ‘moderate’ [35]. Expanding upon this categorical classification method, we computationally weighted these elements. The denominator (${\mathrm{rate}}_{\max}$) reflects the highest possible rating value, in our case, 5 (i.e., scale of 0–5), and serves to balance the equation. $x_{\mathrm{rate}}$ is computed as follows:

$$x_{\mathrm{rate}}=\frac{\mathrm{CI}\left(0.5\right)+\mathrm{nov}\left(0.25\right)+\mathrm{cont}\left(0.25\right)}{{\mathrm{rate}}_{\max}}$$

Calculated $x_{\mathrm{rank}}$ and $x_{\mathrm{rate}}$ scores were imputed into the *QPC* score equation (${\mathrm{QPC}}_{n,k}=x_{\mathrm{rank}}+x_{\mathrm{rate}}$). Following which, *QPC* scores for each question were then summed across participants, creating a *Total Question Priority Composite (Total QPC)* score for each question. The *Total QPC* score is computed as follows:

$$Total\;QPC=\sum_{k=q}^{k=1}{\mathrm{QPC}}_{k}$$

Using this operation, the question with the highest *Total QPC* score is identified as the first priority (i.e., ‘number one’ on the list), the question with the second highest *Total QPC* score is identified as the second priority (i.e., ‘number two’ on the list), and so forth, for all the questions. Questions falling below the tenth‐place position were dropped, resulting in a ranked‐ordered list of the top‐10 priority questions for research addressing the intersections of ABI and MHA. Lastly, to examine whether there were significant differences in *QPC* scores between participants with lived experience and those without, supplemental between‐group analyses (two‐tailed *t*‐tests, *a* = 0.05) were undertaken as well. All analyses were conducted using the statistical computing environment ‘R Studio’ version 1.2.1335.

## 3 Results

Given the stepwise methodological approach of this study, key outcomes for each phase leading to the finalized list of questions for prioritization are reported within the results section. The preliminary literature review yielded 397 articles; after removing duplicates and screening, 135 full texts were evaluated, and as a result, knowledge gaps, recommendations and calls to action were extracted from 36 articles (see Supporting Information S1: Table 2). These raw text excerpts were iteratively synthesized during topic generation, such that redundancies were eliminated and terminology was changed for consistency across research topics (see Supporting Information S1: Table 3). Responses and revisions from the working group were compiled, leading to the generation of a finalized list of priority research topics (see Supporting Information S1: Table 4). During question generation and development, over 80 question fragments from stakeholder responses were coded using over 60 ICF and ICD codes, and a list of 12 research questions was produced and reviewed by the working group. The list contains perspectives and ideas from a broad range of information sources emerging from the extensive procedures described in steps one through five of the priority‐setting methodology.

### 3.1 Question Prioritization

#### 3.1.1 Participants

A total of 59 stakeholders participated in the research question prioritization activity. After removing cases with insufficient data, the final sample included 53 participants. One participant did not consent to the use of their demographic data and therefore was excluded from all demographic analyses. Participants ranged from 22 to 70 years of age (*M* = 46.71 years, SD = 15.13 years). The majority (78%) reported female sex at birth and 80% self‐identified their gender as female (*n* = 3 participants did not disclose sex at birth). Nine (16%) stakeholders reported male sex and gender, and one identified as non‐binary or Indigenous Two‐Spirit. Forty‐one (77%) participants identified as heterosexual, four (8%) identified as homosexual, four (8%) identified as bisexual, one identified as Indigenous Two‐Spirit and two participants did not disclose their sexual orientation. The majority (*n* = 39, 73%) of stakeholders reported their ethnicity as White (British, French, German, North or South American of European background). In total, 17% of stakeholders identified as gender or sexually diverse and 10% self‐reported their ethnicity as Indigenous or mixed‐Indigenous ancestry. The average education level was a bachelor's degree, although stakeholders' levels of educational attainment ranged from less than high school and up to doctoral degrees. Slightly under half (40%) of the sample included people with lived experience. Table 2 is a detailed summary of our sample's sociodemographic characteristics, and Table 3 outlines the participants in our sample organized by type of stakeholder group (e.g., survivor or brain injury, researcher, etc.).

**Table 2:** Participants' sociodemographic characteristics.

Characteristic	*n* (%) of sample^a^
Age (years), mean (SD)	46.71 (15.13)
Sex^b^	
Female	41 (79)
Male	9 (17)
Prefer not to say	2 (4)
Gender^c^	
Female	42 (81)
Male	9 (17)
Non‐binary or Indigenous Two‐Spirit	1 (2)
Sexual orientation	
Heterosexual	41 (79)
Homosexual	4 (8)
Bisexual	4 (8)
Prefer not to say	2 (4)
Indigenous Two‐Spirit	1 (2)
Race or ethnic background	
White (e.g., British, French, German, European background)	39 (75)
East Asian (e.g., Chinese, Korean, Japanese, Taiwanese)	2 (4)
Indigenous Person (e.g., First Nations, Métis, Inuit, Coast Salish)	2 (4)
South Asian (e.g., Indian and Pakistani)	1 (2)
Mixed White and Indigenous	3 (6)
Mixed White and East Asian	1 (2)
Mixed Black and East Asian	1 (2)
Mixed White and Indo‐Caribbean	1 (2)
Prefer not to say	2 (4)
Education	
Less than high school	1 (2)
Some high school	2 (4)
Highschool diploma	4 (8)
Some post‐secondary education	8 (15)
College diploma	10 (19)
Bachelor's degree	19 (37)
Master's degree	5 (10)
Doctoral degree	3 (6)

^a^ One participant did not consent to the collection of their demographic information and is not represented here.

^b^ Sex at birth.

^c^ Current gender identity.

**Table 3:** Participants organized by type of stakeholder and years of experience in their roles.

Type of stakeholder	*n* (%) of sample^a^	Years in role, mean (SD)^b^
Survivor of brain injury	14 (26)	15.92 (9.10)
Family member	11 (21)	15.6 (8.71)
Service provider	18 (34)	7.82 (9.44)
Researcher	7 (13)	4.17 (3.13)
Government representative	3 (6)	19 (14.93)
Public safety worker	3 (6)	5 (2.83)
Health care professional	15 (28)	16.6 (12.79)
Administration	6 (11)	21.4 (9.32)
Occupational therapist (OT)	2 (4)	22 (5.66)
Registered nurse (RN)	1 (2)	38 (n/a)
Physician (MD)	1 (2)	10 (n/a)
Neurorehabilitation consultant	1 (2)	40 (n/a)
Recreation therapist	1 (2)	13 (n/a)
Community support worker	2 (4)	3.5 (0.71)
Case manager	1 (2)	5 (n/a)
Other^c^	2 (4)	Not reported

Abbreviation: SD = standard deviation.

^a^ Many participants identified as more than one type of stakeholder and therefore the number of stakeholder types exceeds the overall sample size.

^b^ Average (mean) number of years in occupation or years affected by ABI (for people with lived experience).

^c^ Other, not specified.

#### 3.1.2 Prioritization Results

**Figure 1:**
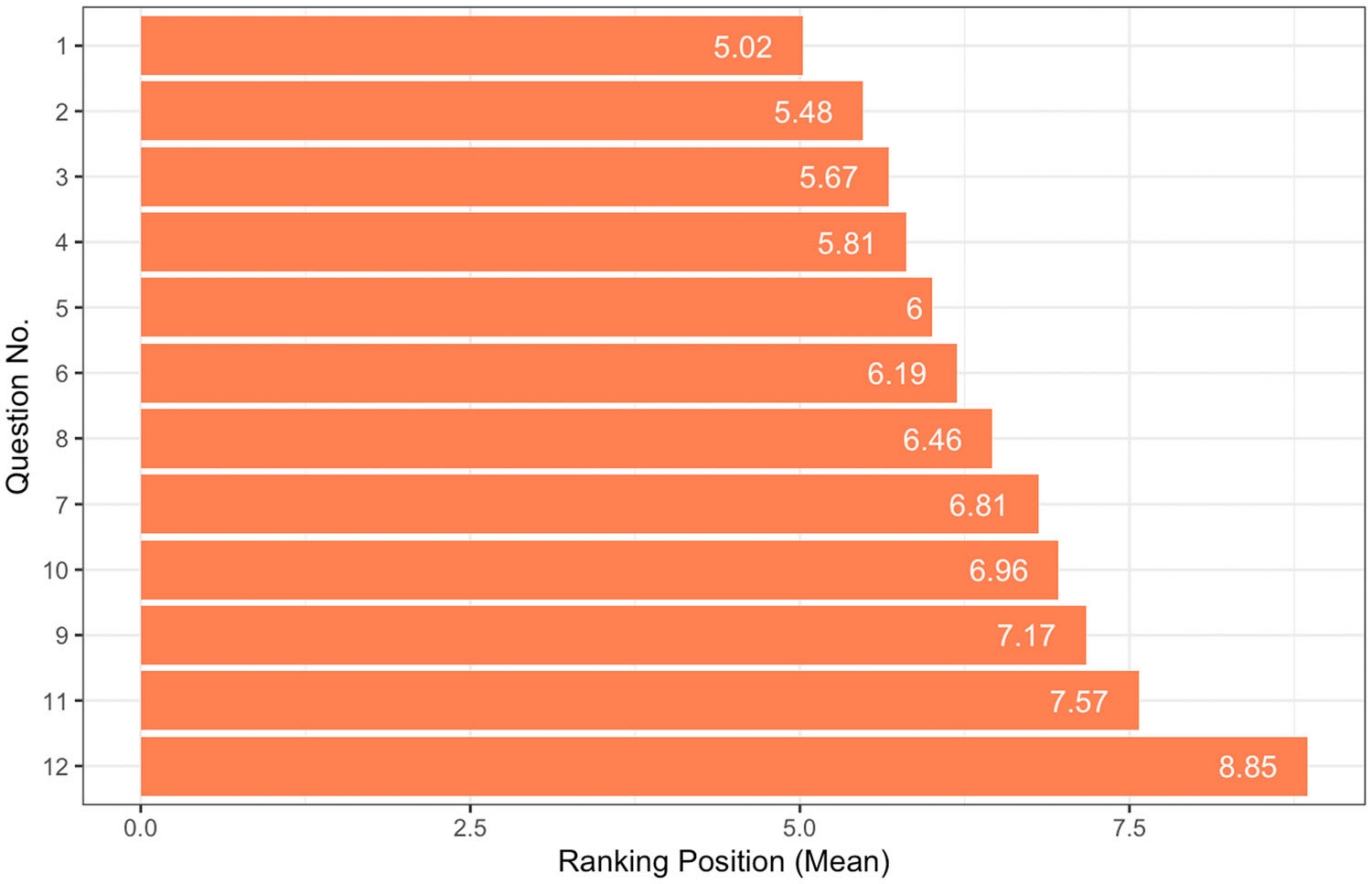
Average ranking position by research question. Question numbers (no.) are labelled here to reflect the order in which they are presented in Table 4, as opposed to the order in which they were originally presented to participants. Values reflect the mean position in which the questions were ranked, with lower values indicating higher priority (i.e., 1 = top priority, 12 = last priority).

**Figure 2:**
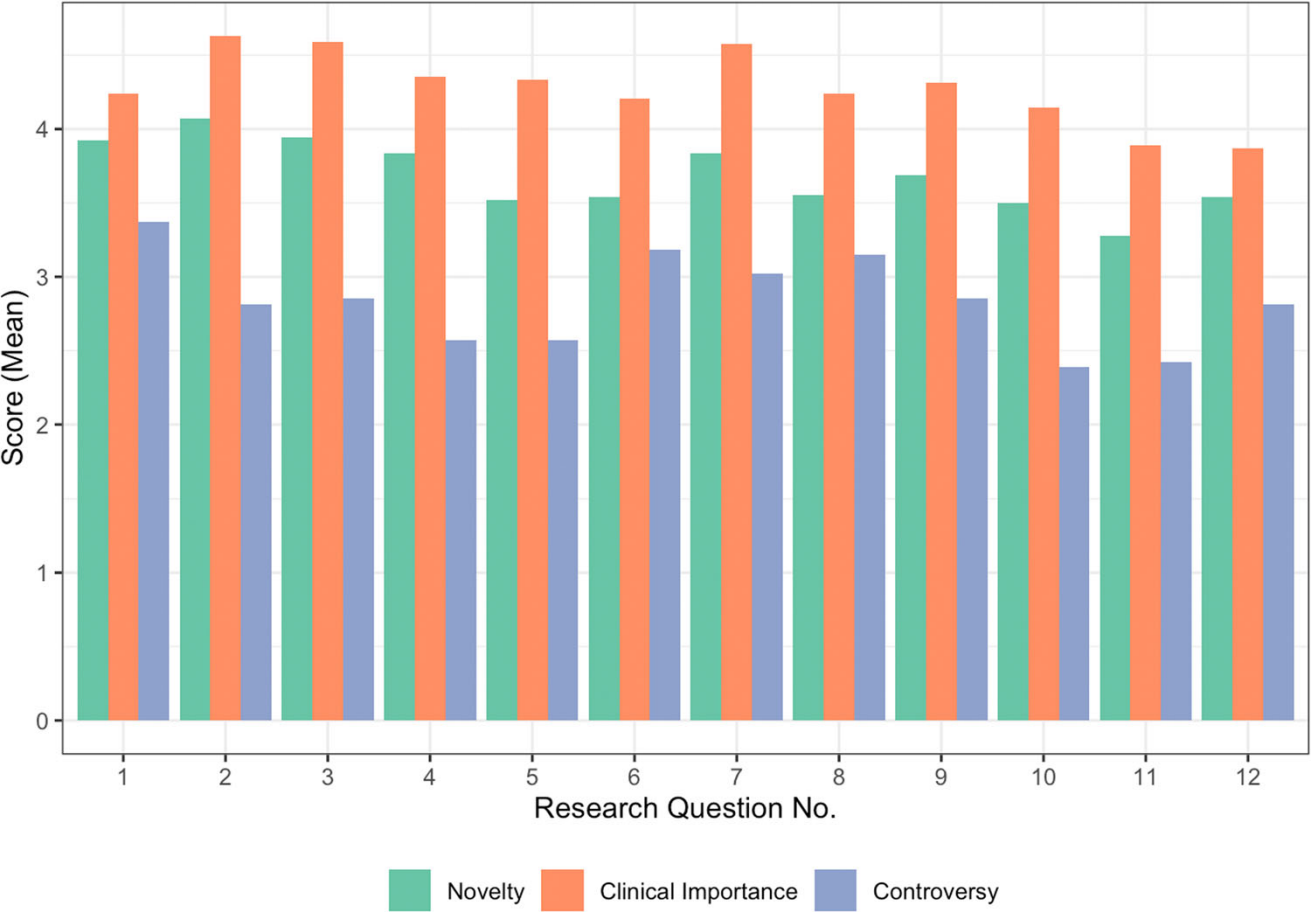
Average scores by rating variable for each research question. Research question numbers (no.) correspond to Table 4.

Question ranking and ratings were analysed and *QPC* scores were calculated. Figure 1 describes the average rank position for each question. Results indicated low levels of concordance between rank positions and questions (*W* = 0.02), suggesting participants' levels of relative priority differed strongly across each of the questions. However, between‐group analyses revealed that participants with lived experience ranked questions more similarly than participants without lived experience (*W* = 0.33, 0.07, respectively). Figure 2 depicts participants' mean ratings of each question across the three variables. On average, clinical importance was rated highest (*M* = 4.28, SD = 0.88), followed by novelty (*M* = 3.70, SD = 1.06), and then controversy (*M* = 2.85, SD = 1.24) across each of the research questions. Results indicated there were no associations between stakeholder groups and rating appraisals (see Supporting Information S1: Table 5), with the exception of novelty for question 10 (*r*_pb_ = 0.31, *p* = 0.025). Lastly, there were no significant between‐group differences in *QPC* scores (*p* > 0.05 for all questions, see Supporting Information S1: Table 6).

The top 10 unanswered research questions addressing the intersections of ABI and MHA are listed in Table 4. Ordered by stakeholders' evaluations of priority, the questions appear by highest to lowest *QPC* scores and reflect stakeholders' combined ranking and rating scores. The two questions with the lowest *QPC* scores, ‘Are “younger” people (e.g., aged 15–30) who experience brain injury at higher risk for developing mood and addiction‐related disorders, compared to middle aged and older adults who experience brain injury?’ and ‘Do personal factors (including age, sex, sexuality, and gender) influence the effectiveness of treatment for people with concurrent brain injury, mental health and addiction disorders?’, were dropped from the list, which resulted in the top 10 research questions. The questions were reviewed by the working group and all unanimously agreed on the outcomes.

**Table 4:** List of the top 10 research questions, rank‐ordered by priority.

No.	Question
1	What are the experiences of concurrent brain injury, mental health and addiction for homeless or marginally housed people, and how do their experiences differ from people who have stable housing?
2	What is the incidence and prevalence of (non‐fatal) opioid overdose‐related hypoxic/anoxic brain injury, and how do we best identify and support people who suffer this type of brain injury?
3	What are the specific long‐term cognitive consequences of (non‐fatal) opioid overdose‐related hypoxic/anoxic brain injury (e.g., problems with attention, memory, communication, etc.)?
4	How do the cognitive (e.g., problems with attention, memory, etc.) and psychosocial (e.g., relationship and family stress, grief, etc.) consequences of brain injury influence survivors' ability to access, engage with and benefit from mental health and addiction treatment services?
5	What are the barriers and facilitators to effective community‐based integrated mental health and addiction treatment for people with concurrent brain injury, mental health and addiction concerns?
6	What are the tools and who is best suited to identify and assess mild‐traumatic brain injury (concussion) in marginalized people struggling with mental health, addiction, trauma and violence?
7	What are the consequences of living with undiagnosed brain injury, and do they include a higher risk of developing mental health and addiction disorders compared to people who receive accurate and timely diagnosis?
8	What is the prevalence of opioid addiction after traumatic brain injury, and what alternative forms of pain management are effective in reducing the risk of opioid addiction after traumatic brain injury?
9	How effective is trauma‐informed counselling for treating people with concurrent brain injury and mental health and addiction disorders, and how does it compare to more traditional mental health and addiction treatments (e.g., alcoholics/narcotics anonymous or behavioural therapies)?
10	What interventions are most effective in promoting quality of life for family members/caregivers of people with concurrent brain injury, mental health and addiction concerns?

## 4 Discussion

The results of this health research priority‐setting process identified the question, ‘What are the experiences of concurrent brain injury, mental health and addiction for homeless or marginally housed people, and how do their experiences differ from people who have stable housing?’ as the top priority for future research. Globally, the disproportionately high prevalence of MHA disorders has been well documented in populations without housing [25]. Far less research has examined ABI in the unhoused. In their systematic review, Stubbs and colleagues [28] identified 22 global studies examining TBI prevalence in homeless and marginally housed individuals. Using random‐effects modelling, the pooled estimate for lifetime history of TBI of any severity, and moderate to severe TBI, was 53.1% and 22.5%, respectively [28]. In a cross‐sectional survey of 500 homeless individuals residing in three major cities in British Columbia, Song et al. [27] found similar prevalence rates, with 63.6% of participants reporting a lifetime history of TBI. Notably, these rates are approximately 2.5 to 4 times for any TBI severity and 10 times higher for moderate to severe TBI than the general population [58]. Others have examined the occurrence and morbidity of cerebrovascular accidents in this population, uncovering similar patterns of disproportionately high rates [59, 60]. Although epidemiological research has been undertaken, far less is known about the lived experiences of concurrent ABI and MHA amongst people who are homeless or marginally housed. Understanding the unique experiences of this vulnerable population may unveil crucial information for removing barriers to effective rehabilitation.

The second and third highest priority research questions both pertain to non‐fatal opioid overdose‐related hypoxic/anoxic (hereafter referred to as hypoxic‐ischaemic) brain injury. Notably, the opioid crisis is an especially pertinent issue in the region where this study was conducted: With over 2500 unregulated drug deaths in 2023 alone, toxic drug poisoning has claimed the lives of over 10,000 British Columbians since the public health emergency declaration was first made in April 2016, making it the province's longest standing public health crisis to date [61]. In cases of non‐fatal drug poisoning events, opioid‐induced respiratory depression can lead to hypoxic‐ischaemic brain injury—a type of ABI caused by restricted oxygen supply—leaving survivors with long‐term mental and physical disability [62]. Certain neurons are especially vulnerable to this type of injury (e.g., hippocampal pyramidal cells and cerebellar purkinje cells) and relate to why learning, memory and spatial coordination deficits are seen amongst survivors [62]. Neuronal damage may also lower self‐awareness, increase disinhibition and reduce self‐efficacy among survivors which, presumably, would make seeking and engaging with treatment exponentially more difficult. However, these assumptions remain un‐investigated.

It is clear that stakeholders in our sample are highly motivated to see researchers examine the interconnections of ABI with substance use and drug poisoning/overdose. Question 8 also alludes to this need, calling for research on the prevalence of opioid dependence after TBI and alternative forms for pain management to reduce the risk of problematic opioid use post‐injury. Chronic pain affects approximately 50% of those with TBI and is commonly treated through opioid prescription receipt [63, 64], heightening the risk of developing opioid use disorder [18, 65]. Several alternative forms of pain management have been researched for chronic pain, but these are yet to be tested amongst TBI patients. The need is urgent, as people with brain injuries are disproportionately represented among those with opioid use disorder [66], and longitudinal data suggests that individuals with a history of TBI are 10 times more likely to die from drug poisoning than the general public [13]. The limited research on these issues demonstrates the urgent need for researchers to take an intersectional approach in their design of studies on ABI and substance use.

Three questions emphasize the importance of ABI recognition and diagnosis. Question 6 is the only one of the ten that focuses specifically on mild TBI. Much of the literature on mild TBI exists within the sports arena, from which we have learned a great deal about the invisibility and diagnostic difficulty unique to this common form of ABI. A growing concern is the role that mild TBI may have in more marginalized groups. For instance, it is estimated that TBI occurs in 50–90% of reported intimate partner violence cases, equating to roughly 250,000 brain injuries each year in Canada [5, 29]. Concerningly, intimate partner violence can also lead to hypoxic‐ischaemic brain injury through strangulation [29], meaning victims are at risk for suffering both traumatic and non‐traumatic forms of ABI. Screening measures have been created and adapted in an effort to increase recognition of intimate partner violence‐related brain injury [29], yet these measures are not widely used in general practice.

Indeed, concurrent MHA concerns, which are inherently high amongst those who experience violence [30], further complicate matters, as post‐concussion symptoms are non‐specific to mild TBI and overlap with common MHA symptoms, such as headache, dizziness, fatigue, depression, anxiety or irritability, making it difficult for both patients and clinicians to disentangle symptomatology and render accurate diagnoses [9, 24, 29, 31]. As question 7 alludes to, it is thought among stakeholders that the consequences of living with undiagnosed ABI might include a higher risk for MHA problems; however, this relationship remains unsubstantiated. Timely and accurate identification of ABI should remain a priority for service providers. Researchers must support these efforts by developing sensitive diagnostic measures for people experiencing concurrent conditions, violence, marginalization and social isolation.

Four of the 10 research priorities are directly aimed at intervention. In relation to question 9, trauma‐informed interventions have been shown to be efficacious for mood, trauma and stress‐related disturbances in the general public [67], but their use after ABI has not been extensively researched. A large body of evidence supports the bi‐directional link between post‐traumatic stress disorder and TBI [68] and, to a lesser extent, non‐traumatic ABI [69]. Specialized trauma‐informed interventions have been developed to target these cooccurrences, but they remain far from widespread clinical implementation [70]. Notably, current clinical practice guidelines do not provide guidance for addressing trauma after ABI [71]. Additionally, studies on how concurrent substance use influences treatment for these groups are limited. Further research is needed given the strong relationship between trauma, substance use and ABI [9, 17, 20, 24, 68].

As question 4 suggests, impaired self‐awareness may decrease survivors' ability to self‐advocate and seek help for MHA concerns [72], while neurocognitive deficits (e.g., impaired planning, organization, memory, and communication) may make it difficult to engage with and maintain treatment [16, 24, 73]. Anecdotal reports of individuals being ‘too brain injured for mental health care’ while ‘too mentally ill for brain injury care’ are common, but researchers have yet to comprehensively formulate and evaluate effective solutions to this real‐world problem. Relating to question 10, this lack of adequate care can lead to family members becoming informal caregivers, the burden of which has been negatively associated with the quality of life for both ABI patients and their caregivers [74, 75]. Question 10 calls for a greater study of interventions for informal caregivers and their potential for promoting quality of life.

Question 5 is arguably the intervention‐focused question with the most previous research. However, the current knowledge base is largely limited to traumatic forms of ABI and strategies are yet to be discovered for integrating care at the population and systems levels [53]. In their systematic review, Chan and colleagues [53] reviewed 15 articles reporting on barriers and/or facilitators to integrated TBI and mental health/substance use care. Barriers such as lack of education, limited access to care and provider hesitancy were contrasted by facilitators, such as education for patients and providers, compensatory strategies for cognitive challenges and family involvement. None of the articles reviewed specifically addressed the needs of underserved or marginalized populations, underscoring the importance of questions 1 and 6. We strongly believe that understanding unique treatment needs and identifying barriers and facilitators to community‐based integrated care for all types of brain injury is the first step in breaking down soiled healthcare services.

### 4.1 Priority Evaluations by Stakeholder Group

While this study sought to understand the collective priorities of stakeholders, our comprehensive analyses provided us the opportunity to explore more nuanced differences in priority evaluations between stakeholder groups. Our results suggested that survivors and family members ranked questions more similarly in comparison to researchers, service providers and other stakeholders without lived experience. Furthermore, we identified a statistically significant relationship between these groups on novelty rating scores for question 10. Interestingly, question 10 is the only one that focuses on family members and caregivers, which may relate to why survivors and family members rated this question as more novel than other stakeholders with less of an ‘insider’, emic perspective. Despite these two small areas of difference, our overall findings suggest that participants had diverse perceptions of priority regardless of their specific stakeholder group membership. This result is to be expected, as the process that led to the creation of the questions produced a list of already high‐priority questions, each of which stakeholders may ascribe a higher degree of priority based on their personal beliefs, experiences and knowledge. These findings underscore the importance of involving a broad range of stakeholders to set priorities that are diversified, accessible and representative.

### 4.2 Methodological Reflections

The methods employed to generate and prioritize these questions were novel such that they built on previous research priority‐setting approaches in two key ways. One, we combined knowledge from multiple sources (i.e., literature and stakeholders) to generate the priority research questions. Two, we are the first to apply a comprehensive ranking and rating strategy to assess stakeholders' evaluations of priority for each question. Although some studies have used both ranking and rating [41], their use of each was sequential and required multiphase surveys of separate samples, demanding more time and resources from participants and research personnel. We believe that our method is more efficient and cost‐effective and that it reduces participation demands by capturing all required data at one time point. In this regard, the creation of the *QPC* formula is one of the most notable strengths. Researchers can use this formula in their own research priority‐setting investigations, across any and all fields of study, to gain a more robust and nuanced understanding of stakeholders' evaluations of priority. We encourage future researchers to utilize the *QPC* method of priority‐setting in their studies and to explore other applications for this unique contribution to the scientific community.

### 4.3 Limitations

This study is not without its limitations. First, the research team are stakeholders in the ABI and MHA communities themselves and regularly interact with other stakeholders, including the working group. While action was taken to promote objectivity through systematic procedures and analyses, it was impossible to eliminate the potential for experimenter bias entirely given our collaborative and community‐engaged approach. Second, people with lived experience may have had personal challenges (e.g., cognitive, physical, psychosocial, or functional difficulties) that made fully engaging with the priority‐setting activity more difficult. To support accessible participation, there was approximately one facilitator for every six participants who were able to provide assistance during the activity. Third, because our sample is primarily from one geographical region, it is probable that their experiences and perspectives may not represent those of stakeholders from other areas of Canada, or the world at large. Instead of setting a broader scope, we chose to focus on meaningful stakeholder engagement and were therefore limited by our means to accommodate in‐person participation. However, our sample size is comparably larger than that of several other priority‐setting studies with in‐person workshops [37, 43, 47]. Lastly, it is our opinion that the priorities identified in this study are not isolated to any specific region, they are in fact global issues that should be addressed worldwide.

It is possible that our findings might not reflect those of people who are actively struggling with the more severe consequences of ABI and MHA, particularly those currently experiencing homelessness. Several actions were taken to promote the inclusion of more marginalized and lower‐income stakeholders, including compensating people with lived experience for their time to participate, as well as covering other costs associated with attending the event, such as travel, accommodation and childcare. While we did not ask participants for information on their income, we did capture their level of education (an indicator of socioeconomic status), which revealed most of the sample had a college diploma at minimum; however, the inclusion of expert researchers and clinicians likely influenced this positive skew. Lastly, although successful in engaging Indigenous and gender and sexually diverse participants, a greater focus on these populations may have revealed unique insights. To better understand the priorities of those most disproportionately affected by the psychosocial consequences of concurrent ABI and MHA, we encourage researchers to build equitable, collaborative relationships with marginalized communities.

## 5 Conclusions

The purpose of this study was to engage a broad range of diverse stakeholders in a health research priority‐setting process to identify, rank and produce a community‐driven list of priorities to guide future research addressing the intersections of ABI and MHA. Our work builds upon previous health research priority‐setting processes by including stakeholders at the earliest stages, evaluating priority through a combination of both ranking and rating assessments, and developing the *QPC* equation to analyse priority appraisals—a unique contribution that can be applied in future priority‐setting studies on any topic—and that people with lived experience made up just under half of our overall sample, a group which has been largely underrepresented in ABI priority‐setting research. To help fill the gap in related literature on marginalized communities, we made notable efforts to involve stakeholders from underserved populations to generate priorities that are equitable and diversified. The results of this study provide researchers with an agenda to focus their efforts on critical and urgent issues currently being experienced by stakeholders. Future research should strive to answer these questions, in addition to producing new and more targeted lines of inquiry as the scientific and stakeholder communities continue to work towards addressing the intersections of brain injury, mental health and addictions.

## Author Contributions

**Cole J. Kennedy:** conceptualization, investigation, methodology, writing–review and editing, writing–original draft, validation, formal analysis, data curation, project administration, funding acquisition. **Erica Woodin:** conceptualization, investigation, funding acquisition, writing–review and editing, supervision. **Julia Schmidt:** conceptualization, investigation, funding acquisition, writing–review and editing, supervision. **Janelle Breese Biagioni:** conceptualization, investigation, funding acquisition, writing–review and editing. **Mauricio A. Garcia‐Barrera:** conceptualization, investigation, funding acquisition, writing–review and editing, methodology, supervision.

## Ethics Statement

Approval for this study was obtained from the University of Victoria (#22‐0614) and the University of British Columbia (#H22‐03403) Human Research Ethics Boards. All individuals who participated in this study provided informed consent.

## Conflicts of Interest

The authors declare no conflicts of interest.

## Supporting information

Supporting information.

## Data Availability

The data that support the findings of this study are available from the corresponding author upon reasonable request. The data are not publicly available due to privacy or ethical restrictions. Ethics approval was not granted for data sharing.
