# Supplementary material for: Ten Priorities for Research Addressing the Intersections of Brain Injury, Mental Health and Addictions: A Stakeholder‐Driven Priority‐Setting Study
Source: Health Expect. 2024 Jul 11;27(4):e14136. doi: 10.1111/hex.14136 (PMC11238575; doi:10.1111/hex.14136)
Supplement: Supplementary file 1 — Supporting information. [file HEX-27-e14136-s001.pdf]

## Supplementary Material

**Supplementary Table 1**. *Peer-reviewed databases and search terms.*

| Database | Search Terms |
|---|---|
| MEDLINE (Ovid) and PsycINFO (EBSCOhost) | “brain injur*” OR “head injur*” OR “traumatic brain injur*” OR “concussion” OR “anoxic brain injur*” OR “hypoxic brain injur*” AND “mental health” OR “mental illness*” OR “mental disorder” OR “psychiatric illness*” OR “psychiatric disorder” AND “substance use” OR “substance use disorder” OR “substance-related disorders” OR “substance abuse” OR “drug use” OR “drug abuse” OR “dependence” OR “addiction*” OR “overdose” OR “opioid-related overdose” |

**Supplementary Table 2.** *Raw text excerpts of identified key recommendations, knowledge gaps, and calls to action.*

*"… sex and gender were often poorly defined in the included studies. Clearly delineating and examining both sex and gender as separate variables is important, as both may have unique roles in the emotional or behavioral impacts of TBI"* (Davies et al., 2022, p. 12).

*"… sex differences in opioid use remain largely unexplored among persons with TBI. This is an important area for future research, as gender or sex can impact outcomes following TBI"* (Adams, 2019, p. 306).

*"The effects of biological sex, as well as gender, which represents an amalgamation of social, biological, cultural and behavioural elements, are underappreciated in traumatic brain injury (TBI) research and practice"* (Mollayeva et al., 2018).

*"…TBI in an older adult sample, which, given the particularly high risk for falls among older MHA service users, represents a significant gap in the current literature"* (Davies et al., 2022, p. 12).

*"Causal relationships between age of TBI and risk of problematic drug and alcohol use later in life is still unknown"* (Cannella, et al., 2019, p. 198).

*"Our findings suggest that more research on the potential moderating effects of sex and age on adolescent TBI outcomes is needed"* (Ilie et al., 2014, p. 13).

*"The non-uniform findings observed bring forth the need to develop and use a comprehensive and consistent methodology in the study of sex and gender post-TBI, incorporating social equity parameters to uncover the potential social underpinnings of gender effects on health and functional outcomes"* (Mollayeva et al., 2021, p. 1).

*"Despite higher rates of violence, this vulnerable population [transgender persons] remains vastly understudied and underserved in the TBI field, which creates health care disparities and limits the ability to deliver appropriate health care to transgender individuals with TBI"* (Giordano et al., 2020, p. 2457).

*"We call for scientists to be trained so they can understand sex and gender influences on their experiments, for health care professionals to be actively mindful about the role TBI can play in relation to sex and gender and vice versa, and finally for improved rehabilitation methods that take sex and gender into account"* (Giordano et al., 2020, p. 2457–2458)

*"… we must consider multiple marginalized identities (e.g., sexuality, education, class, ability, culture, age, and race) in treating individual patients with TBI so quality of life can be maximized"* (Giordano et al., 2020, p. 2458).

**Supplementary Table 2.** *Raw text excerpts of identified key recommendations, knowledge gaps, and calls to action.*

*"Unfortunately, TBI and the effect of sex is understudied and sex-dependent outcomes following TBI remain controversial"* (Cannella, et al., 2019, p. 197).

*"... highlight the need for further research to better understand how females are affected by TBI compared to males, particularly in the context of SUD vulnerability"* (Cannella, et al., 2019, p. 197).

*"Additional research investigating the influence of sex as a biological factor on outcomes following early-life TBI such as SUD vulnerability is critical, particularly among post-pubescent adolescent patients where fluctuating hormone levels can be taken into consideration. Further studies will improve the understanding and awareness of sex-specific vulnerabilities to TBI and interactions with persistent psychiatric problems like SUD and will advance individual strategies for treatment"* (Cannella, et al., 2019, p. 198).

*"Previous studies have documented an increased risk of injury attributable to victimization or risk-taking behavior among sexual minority homeless youths, and these finding may suggest that this increased risk also applies to TBI"* (Mackelprang et al., 2014, p. 1989).

*"Complexities of needs present in some adults with TBI suggest the need for comprehensive assessment and tailored treatment across a variety of service settings. This again supports the critical importance of approaching integrated management of TBI from an interdisciplinary perspective"* (Coxe et al., 2021, p. 113).

*"... there is a dearth of evidence regarding suicide prevention interventions for those with a history of TBI. Further research aimed at identifying such evidence-based interventions is needed"* (Schneider et al., p. 4).

*"Further investigation into the reason for a lack of studies and efforts to contribute evidence to this body of literature is warranted to inform how integrated care might be implemented, including barriers and facilitators to integrated care, or how effective that care might be for individuals with TBI and MHSU at the population level"* (Chan et al., 2022, p. 13).

*"Future studies should also examine assessment and referral strategies to increase low substance use treatment engagement among patients with TBI, as well as potential moderating factors such as accessibility barriers"* (VanderVeen, 2021, p. 1207).

*"Future research is needed to better understand how cross-system collaboration can increase TBI implementation practices from the perspectives of the clients, family members, and social services and medical providers"* (Coxe et al., 2021, p. 287).

*"Further research on rehabilitation after intentional TBI could inform more focused prevention and treatment strategies, as well as provide ongoing evaluation of such strategies"* (Kim et al., 2013, p. 2219).

**Supplementary Table 2.** *Raw text excerpts of identified key recommendations, knowledge gaps, and calls to action.*

*"Community-based treatment for substance abuse for individuals with TBI is required. Inpatient or residential treatment for persons with TBI is seldom available or affordable"* (Corrigan, 2005).

*"The importance and timeliness of elucidating unique risk factors, barriers to treatment, prevention opportunities, and treatment accommodations for those with TBI at risk for OUD cannot be understated"* (Adams et al., 2019, p. 213).

*"The present review highlights the importance of understanding pre-TBI substance use behaviors prior to administering substance use interventions. More work is needed to understand factors affecting treatment engagement and outcomes in this population"* (VanderVeen, 2021, p. 1207).

*"The current study also underscores the need for validated methods of identifying aspects of a prior history of TBI"* (Corrigan & Deutschle, 2008, p. 230).

*"Research on screening and brief intervention for persons with TBI is a case in point that demonstrates how treatment can be improved"* (Corrigan, 2021, p. 1062).

*"Psychometrically sound screening tools available to aid in the identification of lifetime history of traumatic brain injury (TBI) are limited"* (Schneider et al., 2016, p. 1).

*"Additional work is necessary to assess the relations between screening questions and outcomes of interest… Further research is certainly required to assess the potential positive or negative impact of comprehensive TBI screening in MH settings"* (Brenner et al., 2013, p. 26–29).

*"Study findings suggest the need for accurate assessment to identify those at greatest risk for problematic substance use behaviors after TBI"* (VanderVeen, 2021, p. 1198).

*"…two recent studies [(Iverson et al., 2005; Lange et al., 2008)] found that commonly used assessment tools could not distinguish between CI [cognitive impairment] from mild traumatic brain injury and CI from SUD [substance use disorder]"* (Hill & Colistra, 2014, p. 21).

*"Screening for TBI in these [mental health/addiction] settings may provide insight into behaviors or ability to engage with care"* (Davies et al., 2022, p. 13).

**Supplementary Table 2.** *Raw text excerpts of identified key recommendations, knowledge gaps, and calls to action.*

*"Future research is also needed to examine how organizational contextual factors as well as characteristics of the TBI screening methods and accommodations may influence implementation of TBI care within SUD treatment organizations"* (Coxe et al., 2021, p. 287).

*"The behavioral health workforce needs to secure the knowledge and skills to identify patients' problematic histories of TBI and accommodate the resulting neurological effects in treatment"* (Corrigan 2021, p. 1057).

*"Developing interventions to address difficulties with depression among brain injury survivors should be a priority, and those interventions should be tailored to meet the challenges of access to care (e.g., transportation issues) and concomitant difficulties often present following TBI (e.g., cognitive difficulties)"* (Mackelprang et al., 2014a, p. 105)

*"Such individuals [TBI patients] may also benefit from treatment modifications (e.g., shorter sessions, slower pace) and interventions focused on psychosocial functioning"* (Brenner et al., 2013, p. 27).

*"Cognitive consequences of brain damage such as impaired decision-making and reduced self-awareness have been speculated to make it more difficult for patients to successfully engage in addiction treatment"* (Davies et al., 2022, p. 12).

*"For a subset of individuals with TBI, cognitive deficits (e.g., executive dysfunction) may combine with maladaptive coping and poor psychosocial adjustment to increase risk for the development of chronic substance use/ misuse"* (Zgaljardic et al., 2015, p. 1918).

*"There are multiple opportunities to study how proven behavioral health interventions may need to be modified to better serve persons with TBI"* (Corrigan, 2021, p. 1062).

*"TBI-related deficits in planning, memory, or decision-making could warrant modifications to treatment modalities that require increased cognitive engagement such as cognitive behavioral therapy, though robust assessment of modified interventions has been limited thus far"* (Davies et al., 2022, p. 12).

*"… studies on how TBI affects addiction vulnerability are lacking"* (Cannella, et al., 2019, p. 196).

*"Further studies are required to collect data on the temporal relationship of onset of TBI and the diagnosis of BD investigating if BD could predispose to TBI"* (Bacciardi et al. 2017, p. 44)

**Supplementary Table 2.** *Raw text excerpts of identified key recommendations, knowledge gaps, and calls to action.*

*"Impaired attention and concentration affect SUD treatment in multiple ways… clients require memory supports to reliably engage in treatment… Impaired executive functioning can result in SUD clients having difficulty naming goals… This recognizes that treating cognitively impaired SUD clients requires some specialization and adaptation of SUD treatment strategies"* (Hill & Colistra, 2014, p. 26–29).

*"Cognitive impairment is a common feature of individuals with substance-use disorders. However, research tends to exclude highly complex clinical cases, limiting the generalisability of findings for 'real-world' populations… there is a need for comprehensive, specialist neuropsychological assessment in addiction settings"* (Gooden et al., 2021, p. 83–90).

*"Effective TBI preventive programs among youth should be initiated to help reduce the incidence of TBI and with it the development of substance misuse problems that may develop post TBI"* (Ilie et al., 2015, p. 15–16).

*"As adolescents 13 years and older are presenting to healthcare settings for TBI at a rapidly increasing rate, and adolescence and emerging adulthood represent the peak age of onset for most mental illnesses, further exploration of TBI among youth MHA [mental health addiction] service users is warranted"* (Davies et al., 2022, p. 12).

*"Furthermore, there is evidence to suggest that mood difficulties such as frustration, irritability, stress, attention, and worry can arise as a result of PPCS. However, minimal research has focused on this area. Both adolescents and adults suffer from PPCS, but literature on adolescents appears to be lacking compared to adults"* (Sheldrake et al., 2022, p. 12).

*"… the paucity of relevant research into long-term psychiatric consequences following sport concussion in adults is initially surprising, especially given the increased attention paid by the media and sports organizations"* (Finkbeiner et al., 2016, p. 275).

*"To better understand the relationship between pre-TBI factors, TBI, and subsequent antisocial behavior, more work is needed. In turn, future research can inform the legal system, target rehabilitation efforts, and help policy makers better grasp the costs of TBI in society at large"* (Elbogen et al., 2015, p. 12).

*"Substance use is one of the leading predictors of sustaining a TBI, and the vast majority of all TBIs sustained are mild. Therefore, it is important to appropriately screen for substance use behaviors in the acute care setting and deliver targeted treatment to those with higher use rates because even maintaining use rates after injury puts people at risk for future injury and negative outcomes… More longitudinal work is needed to assess substance use behavior trajectories over a longer period of time after TBI, particularly in child and adolescent samples"* (VanderVeen, 2021, p. 1206–1207).

**Supplementary Table 2.** *Raw text excerpts of identified key recommendations, knowledge gaps, and calls to action.*

*"Substance abuse prevention efforts should be directed toward adolescents who have experienced multiple head injuries given that this subpopulation is more likely to experience cognitive impairments that influence riskier forms of behavior"* (Veliz et al., 2021, p. 190).

*"Traumatic brain injury (TBI) and mental health and/or substance use challenges (MHSU) are commonly co-occurring and prevalent in individuals experiencing homelessness; however, evidence suggests that systems of care are siloed and organized around clinical diagnoses. Research is needed to understand how housing and housing supports are provided to this complex and understudied group in the context of siloed service systems"* (Estrella et al., 2021, p. 1).

*"The findings of this study support and unpack concerns raised by other researchers regarding the limited knowledge on TBI among those working in housing and the practices of moving individuals with co-occurring conditions to systems and services that are perceived to better serve them…Given that research has tended to study those with TBI and MHSU in isolation from one another, there may be benefits in future research that brings together knowledge from both fields and applying it to the issue of housing"* (Estrella et al., 2021, p. 15).

*"Future research is necessary to determine the most effective ways of identifying homeless individuals who have sustained TBIs and connecting them to appropriate services that will promote community integration"* (Mackelprang et al., 2014b, p. 1991).

*"Effective interventions and policies to reduce the risk of TBI among people experiencing homelessness are needed, and the provision of housing is a promising approach to achieve this goal"* (Mejia-Lancheros et al., 2020, p. 5).

*"Establishing whether TBI is a risk factor for poor outcomes (eg, homelessness or serious health conditions) will be important to understand and address the impact of TBI in this population… High-quality studies are urgently needed to elucidate the true prevalence and incidence of TBI, and the directionality of the relationship between TBI and outcomes in the homeless and marginally housed population"* (Stubbs et al., 2020, p. 30).

*"Neurocognitive sequelae from opioid overdoses in humans have not been rigorously or systematically studied"* (Winstanley et al., 2021, p. 2).

*"…the paucity of findings on the chronic complications resulting from recurring non-fatal opioid overdose events are alarming given the evidence we do have on hypoxic brain injuries stemming from opioid-induced toxicity… A gap also appears to exist on whether* existing *physical and mental health outcomes among persons who misuse and abuse opioids become exacerbated when said*

**Supplementary Table 2.** *Raw text excerpts of identified key recommendations, knowledge gaps, and calls to action.*

*consumers experience (>1) non-fatal opioid overdose"* (Zibbell et al., 2019, p. 13).

*"Yet, to date, there is no systematic empirical evidence on the incidence of overdose-related neurocognitive impairments and no definitive evidence stating whether or not neurocognitive impairments that result in poor treatment outcomes are attributable to overdose"* (Winstanley et al., 2021, p. 36)

*"Additional research is needed to understand long-term implications of serious opioid overdoses and their impact on people's quality of life"* (Kitchen et al., 2021, p. 5).

*"Rigorously designed prospective case control studies, that control for confounding factors, are needed to empirically measure changes in the brain and cognition that occur following opioid-related overdose"* (Winstanley et al., 2021, p. 36).

*"To date, there are no known studies examining experiences or outcomes of persons with TBI seeking treatment specifically for OUD… More research is needed to understand patterns of prescription opioid use and associated outcomes following TBI for those with and without preinjury opioid use… More research is needed to fully understand the association of high-risk opioid use with adverse outcomes among TBI survivors"* (Adams, 2019, p. 304–307).

*"The results reported are not yet sufficient to draw conclusive evidence on the incidence, magnitude, and consequences of opioid overdose-related brain injuries. Therefore, further investigation into these types of impairments is warranted"* (Winstanley et al., 2021, p. 36).

*"There were no studies evaluating interventions to reduce use of opioids in TBI populations. Preliminary findings suggest prescription opioid receipt is strongly related to psychological symptoms, including comorbid depression, anxiety, and post-traumatic stress disorder… Future studies should include more varied patient populations as well as evaluate interventions to reduce opioid use following TBI"* (Starosta et al., 2021, p. 1–2).

*"More study is needed to identify non-pharmacological approaches to treating pain among people with TBI. More research is warranted to examine whether a history of TBI increases risk for OUD, including among individuals with mild TBI in which symptoms appear to have resolved, and to determine if additional barriers exist for people with TBI and OUD to access evidence-based treatments"* (Corrigan & Adams, 2019, p. 144).

*"Prevalence and consequences of OD-related brain injuries is unknown"* (Winstanley et al., 2021, p. 1).

**Supplementary Table 2.** *Raw text excerpts of identified key recommendations, knowledge gaps, and calls to action.*

*"Most work that focuses on prevalence rates and substance-use treatment continues to focus on alcohol, with less attention to other addictive substances. As opiate use is common in head-injured individuals, many of whom experience severe and often chronic pain, it is surprising that so little about opiate addiction risks is reported"* (Graham & Cardon, 2008, p. 159).

*"Time with inadequate respiration likely predicts the extent of brain injuries and it is unknown if that critical information is systematically captured in the patients' electronic medical records…Clinicians may want to consider screening for acute brain injuries and/or neurocognitive impairments in individuals known to have experienced a prolonged period of hypoxia or anoxia due to an opioid overdose and consider monitoring for delayed onset for those with the highest risk…The incidence and prevalence of opioid-related brain injuries is needed to inform clinical care and post overdose management, particularly in terms of whether screening for brain injuries is warranted in this population"* (Winstanley et al., 2021, p. 36).

**Note:** Extracted recommendations, knowledge gaps, and calls to action are approximately ordered in relation to their corresponding preliminary research topics, as listed in Supplementary Table 3. See Supplementary References (p. 14–16) for full list of sources.

**Supplementary Table 3.** *Preliminary list of research topics.*

1. Understanding the influence of age, sex, sexuality, and gender on prevention measures, treatment approaches, neurological vulnerabilities, and long-term outcomes in individuals with concurrent brain injury and mental health/addiction disorders.

2. Identifying barriers and facilitators to effective community-based integrated treatment services for individuals with concurrent brain injury and mental health/addiction concerns.

3. Understand the neurological, cognitive, and behavioural consequences of non-fatal opioid overdose-related hypoxic/anoxic brain injury.

4. Creating tools and best practice guidelines for screening for brain injury in mental health/addictions settings, and screening for mental health/addiction disorders in brain injury rehabilitation settings.

5. Understanding how the neurobehavioral consequences of brain injury (e.g., problems with attention, memory, executive function, etc.) affect survivors' ability to access, engage with, and benefit from mental health/addiction treatment services.

6. Developing effective mild-traumatic brain injury (concussion) screening and assessment tools for use in marginalized populations struggling with mental health/addiction, trauma, violence, and homelessness.

7. Understanding risky behaviours (e.g., substance use, criminality, etc.), mental health/addiction, neuropsychological problems (e.g., attention, memory, impulse control, etc.), and risk for re-injury in young adults with mild-traumatic brain injury (concussion).

8. Understanding brain injury in individuals who are homeless or marginally housed as it intersects with mental health/addiction and overdose.

9. Examining outcomes of opioid prescription for persons with traumatic brain injury and evaluating interventions to reduce long-term misuse of opioids in persons with traumatic brain injury.

10. Developing methods, measures, and best practice guidelines for accurately identifying and treating hypoxic/anoxic brain injury following non-fatal opioid-related overdose.

**Note:** The topics are listed here in no particular order.

**Supplementary Table 4.** *Finalized list of research topics used in question generation.*

1. Understanding the influence of personal factors such as age, sex, sexuality, and gender on treatment effectiveness and long-term outcomes of individuals with concurrent brain injury and mental health/addiction disorders.

2. Identifying barriers and facilitators to effective community-based integrated treatment services for individuals with concurrent brain injury and mental health/addiction concerns.

3. Understanding the neurological, cognitive, and behavioural consequences of non-fatal opioid overdose-related hypoxic/anoxic brain injury.

4. Understanding how the neurobehavioral consequences of brain injury (e.g., problems with attention, memory, language, etc.) affect survivors' ability to access, engage with, and benefit from mental health/addiction treatment services.

5. Developing effective mild-traumatic brain injury (concussion) assessment tools and best practice guidelines for use in marginalized populations struggling with mental health/addiction, trauma, and violence.

6. Understanding brain injury in individuals who are homeless or marginally housed as it intersects with mental health/addiction and overdose.

7. Evaluating interventions to reduce long-term misuse of opioids in persons with traumatic brain injury, including examining outcomes of opioid prescription.

8. Developing methods, measures, and best practice guidelines for accurately identifying and treating hypoxic/anoxic brain injury (i.e., temporary partial or total lack of oxygen to the brain) following non-fatal opioid-related overdose.

**Note:** The topics are listed here in no particular order.

**Supplementary Table 5.** *Point-biserial result for rating variables and participant groups.*

| Variable | $r_{pb}$ | *p*-value |
|---|---|---|
| Clinical Importance | ------- | ------- |
| Question 1 | -0.129 | .353 |
| Question 2 | -0.061 | .660 |
| Question 3 | -0.088 | .527 |
| Question 4 | 0.042 | .764 |
| Question 5 | -0.013 | .925 |
| Question 6 | -0.027 | .846 |
| Question 7 | -0.083 | .550 |
| Question 8 | 0.065 | .639 |
| Question 9 | -0.048 | .731 |
| Question 10 | 0.127 | .360 |
| Novelty | ------- | ------- |
| Question 1 | -0.089 | .520 |
| Question 2 | 0.101 | .466 |
| Question 3 | 0.132 | .343 |
| Question 4 | 0.103 | .457 |
| Question 5 | -0.099 | .475 |
| Question 6 | 0.141 | .310 |
| Question 7 | -0.190 | .169 |
| Question 8 | -0.062 | .658 |
| Question 9 | 0.200 | .147 |
| Question 10 | 0.305 | .025* |
| Controversary | ------- | ------- |
| Question 1 | -0.071 | .611 |
| Question 2 | -0.190 | .169 |
| Question 3 | -0.142 | .307 |
| Question 4 | -0.085 | .540 |
| Question 5 | -0.095 | .495 |
| Question 6 | -0.139 | .315 |
| Question 7 | -0.017 | .902 |
| Question 8 | -0.103 | .459 |
| Question 9 | -0.174 | .210 |
| Question 10 | -0.061 | .662 |

**Note:** *Indicates significance, $p < .05$. Groups include participants with lived experience and participants without lived experience. Research question numbers correspond to Table 4 of the main text.

**Supplementary Table 6.** *QPC means, standard deviations, t-statistics, and p-values by group.*

| | No Lived Experience (*n* = 31) | | Lived Experience (*n* = 21) | | | |
|---|---|---|---|---|---|---|
| | Mean | SD | Mean | SD | t-statistic | p-value |
| Question 1 | 1.49 | 0.25 | 1.38 | 0.35 | 1.35 | 0.184 |
| Question 2 | 1.42 | 0.35 | 1.45 | 0.31 | -0.40 | 0.691 |
| Question 3 | 1.35 | 0.33 | 1.48 | 0.23 | -1.73 | 0.090 |
| Question 4 | 1.40 | 0.26 | 1.37 | 0.33 | 0.32 | 0.754 |
| Question 5 | 1.33 | 0.36 | 1.37 | 0.36 | -0.40 | 0.690 |
| Question 6 | 1.30 | 0.33 | 1.31 | 0.31 | -0.06 | 0.952 |
| Question 7 | 1.27 | 0.29 | 1.35 | 0.37 | -0.88 | 0.385 |
| Question 8 | 1.29 | 0.34 | 1.28 | 0.34 | 0.07 | 0.947 |
| Question 9 | 1.28 | 0.38 | 1.29 | 0.33 | -0.10 | 0.918 |
| Question 10 | 1.20 | 0.41 | 1.23 | 0.40 | -0.32 | 0.752 |

Groups include participants with lived experience and participants without lived experience. Research question numbers correspond to Table 4 of the main text.

**Supplementary References**

Adams, R. S., Corrigan, J. D., & Dams-O'Connor, K. (2020). Opioid Use among Individuals with Traumatic Brain Injury: A Perfect Storm? *Journal of Neurotrauma*, *37*(1), 211–216. https://doi.org/10.1089/neu.2019.6451

Brenner, L. A., Homaifar, B. Y., Olson-Madden, J. H., Nagamoto, H. T., Huggins, J., Schneider, A. L., Forster, J. E., Matarazzo, B., & Corrigan, J. D. (2013). Prevalence and screening of traumatic brain injury among veterans seeking mental health services. *The Journal of Head Trauma Rehabilitation*, *28*(1), 21–30. https://doi.org/10.1097/HTR.0b013e31827df0b5

Cannella, L. A., McGary, H., & Ramirez, S. H. (2019). Brain Interrupted: Early Life Traumatic Brain Injury and Addiction Vulnerability. *Experimental Neurology*, *317*, 191–201. https://doi.org/10.1016/j.expneurol.2019.03.003

Chan, V., Toccalino, D., Omar, S., Shah, R., & Colantonio, A. (2022). A systematic review on integrated care for traumatic brain injury, mental health, and substance use. *PloS One*, *17*(3), e0264116. https://doi.org/10.1371/journal.pone.0264116

Corrigan, J. D. (2005). Substance Abuse. In *Rehabilitation for traumatic brain injury* (pp. 133–155). Oxford University Press.

Corrigan, J. D. (2021). Traumatic Brain Injury and Treatment of Behavioral Health Conditions. *Psychiatric Services*, *72*(9), 1057–1064. https://doi.org/10.1176/appi.ps.201900561

Corrigan, J. D., & Adams, R. S. (2019). The intersection of lifetime history of traumatic brain injury and the opioid epidemic. *Addictive Behaviors*, *90*, 143–145. https://doi.org/10.1016/j.addbeh.2018.10.030

Corrigan, J. D., & Deutschle Jr, J. J. (2008). The presence and impact of traumatic brain injury among clients in treatment for co-occurring mental illness and substance abuse. *Brain Injury*, *22*(3), 223–231. https://doi.org/10.1080/02699050801938967

Coxe, K. A., Pence, E. K., & Kagotho, N. (2021). Social Work Care in Traumatic Brain Injury and Substance Use Disorder Treatment: A Capacity-Building Model. *Health & Social Work*, *46*(4), 277–288. https://doi.org/10.1093/hsw/hlab023

Davies, J., Dinyarian, C., Wheeler, A. L., Dale, C. M., & Cleverley, K. (2023). Traumatic Brain Injury History Among Individuals Using Mental Health and Addictions Services: A Scoping Review. *Journal of Head Trauma Rehabilitation*, *38*(1), E18–E32. https://doi.org/10.1097/HTR.0000000000000780

Elbogen, E. B., Wolfe, J. R., Cueva, M., Sullivan, C., & Johnson, J. (2015). Longitudinal Predictors of Criminal Arrest After Traumatic Brain Injury: Results From the Traumatic Brain Injury Model System National Database. *Journal of Head Trauma Rehabilitation*, *30*(5), E3–E13. https://doi.org/10.1097/HTR.0000000000000083

Estrella, M. J., Kirsh, B., Kontos, P., Grigorovich, A., Colantonio, A., Chan, V., & Nalder, E. J. (2021). Critical Characteristics of Housing and Housing Supports for Individuals with Concurrent Traumatic Brain Injury and Mental Health and/or Substance Use Challenges: A Qualitative Study. *International Journal of Environmental Research and Public Health*, *18*(22), 12211. https://doi.org/10.3390/ijerph182212211

Finkbeiner, N. W. B., Max, J. E., Longman, S., & Chantel, D. (2016). Knowing What We Don't Know: Long-Term Psychiatric Outcomes following Adult Concussion in Sports. *The Canadian Journal of Psychiatry*, *61*(5), 270-276.

Giordano, K. R., Rojas-Valencia, L. M., Bhargava, V., & Lifshitz, J. (2020). Beyond Binary: Influence of Sex and Gender on Outcome after Traumatic Brain Injury. *Journal of Neurotrauma*, *37*(23), 2454–2459. https://doi.org/10.1089/neu.2020.7230

Gooden, J. R., Cox, C. A., Petersen, V., Curtis, A., Manning, V., & Lubman, D. I. (2021). Characterisation of presentations to a community-based specialist addiction neuropsychology service: Cognitive profiles, diagnoses and comorbidities. *Drug and Alcohol Review*, *40*(1), 83–92. https://doi.org/10.1111/dar.13135

Graham, D. P., & Cardon, A. L. (2008). An Update on Substance Use and Treatment following Traumatic Brain Injury. *Annals of the New York Academy of Sciences*, *1141*(1), 148–162. https://doi.org/10.1196/annals.1441.029

Hill, T. K., & Colistra, A. L. (2014). Addiction-Related Cognitive Impairment in Substance Use Disorder Treatment: Behavioral Suggestions for Addictions Treatment Practitioners. *Alcoholism Treatment Quarterly*, *32*(1), 19–32. https://doi.org/10.1080/07347324.2013.831688

Ilie, G., Adlaf, E. M., Mann, R. E., Boak, A., Hamilton, H., Asbridge, M., Colantonio, A., Turner, N. E., Rehm, J., & Cusimano, M. D. (2014). The Moderating Effects of Sex and Age on the Association between Traumatic Brain Injury and Harmful Psychological Correlates among Adolescents. *PLOS ONE*, *9*(9), e108167. https://doi.org/10.1371/journal.pone.0108167

Ilie, G., Mann, R. E., Boak, A., Adlaf, E. M., Hamilton, H., Asbridge, M., Rehm, J., & Cusimano, M. D. (2014). Suicidality, Bullying and Other Conduct and Mental Health Correlates of Traumatic Brain Injury in Adolescents. *PLOS ONE*, *9*(4), e94936. https://doi.org/10.1371/journal.pone.0094936

Kim, H., Bayley, M., Dawson, D., Mollayeva, T., & Colantonio, A. (2013). Characteristics and functional outcomes of brain injury caused by physical assault in Canada: A population-based study from an inpatient rehabilitation setting. *Disability and Rehabilitation*, *35*(26), 2213–2220. https://doi.org/10.3109/09638288.2013.774063

Kitchen, S. A., McCormack, D., Werb, D., Caudarella, A., Martins, D., Matheson, F. I., & Gomes, T. (2021). Trends and outcomes of serious complications associated with non-fatal opioid overdoses in Ontario, Canada. *Drug and Alcohol Dependence*, *225*, 108830. https://doi.org/10.1016/j.drugalcdep.2021.108830

Mackelprang, J. L., Bombardier, C. H., Fann, J. R., Temkin, N. R., Barber, J. K., & Dikmen, S. S. (2014a). Rates and Predictors of Suicidal Ideation During the First Year After Traumatic Brain Injury. *American Journal of Public Health*, *104*(7), e100–e107. https://doi.org/10.2105/AJPH.2013.301794

Mackelprang, J. L., Harpin, S. B., Grubenhoff, J. A., & Rivara, F. P. (2014b). Adverse Outcomes Among Homeless Adolescents and Young Adults Who Report a History of Traumatic Brain Injury. *American Journal of Public Health*, *104*(10), 1986–1992. https://doi.org/10.2105/AJPH.2014.302087

Mejia-Lancheros, C., Lachaud, J., Stergiopoulos, V., Matheson, F. I., Nisenbaum, R., O'Campo, P., & Hwang, S. W. (2020). Effect of Housing First on violence-related traumatic brain injury in adults with experiences of homelessness and mental illness: Findings from the At Home/Chez Soi randomised trial, Toronto site. *BMJ Open*, *10*(12), e038443. https://doi.org/10.1136/bmjopen-2020-038443

Mollayeva, T., Mollayeva, S., & Colantonio, A. (2018). Traumatic brain injury: Sex, gender and intersecting vulnerabilities. *Nature Reviews Neurology*, *14*(12), 711–722. https://doi.org/10.1038/s41582-018-0091-y

Mollayeva, T., Mollayeva, S., Pacheco, N., & Colantonio, A. (2021). Systematic Review of Sex and Gender Effects in Traumatic Brain Injury: Equity in Clinical and Functional Outcomes. *Frontiers in Neurology*, *12*, 678971. https://doi.org/10.3389/fneur.2021.678971

Schneider, A. L., Hostetter, T. A., Homaifar, B. Y., Forster, J. E., Olson-Madden, J. H., Matarazzo, B. B., Huggins, J., & Brenner, L. A. (2016). Responses to Traumatic Brain Injury Screening Questions and Suicide Attempts among Those Seeking Veterans Health Administration Mental Health Services. *Frontiers in Psychiatry*, *7*, 59. https://doi.org/10.3389/fpsyt.2016.00059

Sheldrake, E., Al-Hakeem, H., Lam, B., Goldstein, B. I., Wheeler, A. L., Burke, M., Dunkley, B. T., Reed, N., & Scratch, S. E. (2022). Mental Health Outcomes Across the Lifespan in Individuals With Persistent Post-Concussion Symptoms: A Scoping Review. *Frontiers in Neurology*, *13*. https://doi.org/10.3389/fneur.2022.850590

Silvia Bacciardi, Angelo G.I. Maremmani, Nooshin Nikoo, Luca Cambioli, Christian Schütz, Kerry Jang, & Michael Krausz. (2017). Is bipolar disorder associated with tramautic brain injury in the homeless? *Rivista Di Psichiatria*, *52*(1), 40–56. https://doi.org/10.1708/2631.27053

Starosta, A. J., Adams, R. S., Marwitz, J. H., Kreutzer, J., Monden, K. R., Dams O'Connor, K., & Hoffman, J. (2021). Scoping Review of Opioid Use After Traumatic Brain Injury. *The Journal of Head Trauma Rehabilitation*, *36*(5), 310. https://doi.org/10.1097/HTR.0000000000000721

Stubbs, J. L., Thornton, A. E., Sevick, J. M., Silverberg, N. D., Barr, A. M., Honer, W. G., & Panenka, W. J. (2020). Traumatic brain injury in homeless and marginally housed individuals: A systematic review and meta-analysis. *The Lancet Public Health*, *5*(1), e19–e32. https://doi.org/10.1016/S2468-2667(19)30188-4

VanderVeen, J. D. (2021). TBI as a Risk Factor for Substance Use Behaviors: A Meta-analysis. *Archives of Physical Medicine and Rehabilitation*, *102*(6), 1198–1209. https://doi.org/10.1016/j.apmr.2020.10.112

Veliz, P., McCabe, S. E., Eckner, J. T., & Schulenberg, J. E. (2021). Concussion, Sensation-Seeking and Substance use among US Adolescents. *Substance Abuse*, *42*(2), 183–191. https://doi.org/10.1080/08897077.2019.1671938

Winstanley, E. L., Mahoney, J. J., Castillo, F., & Comer, S. D. (2021). Neurocognitive impairments and brain abnormalities resulting from opioid-related overdoses: A systematic review. *Drug and Alcohol Dependence*, *226*, 108838. https://doi.org/10.1016/j.drugalcdep.2021.108838

Zgaljardic, D. J., Seale, G. S., Schaefer, L. A., Temple, R. O., Foreman, J., & Elliott, T. R. (2015). Psychiatric Disease and Post-Acute Traumatic Brain Injury. *Journal of Neurotrauma*, *32*(23), 1911–1925. https://doi.org/10.1089/neu.2014.3569

Zibbell, J., Howard., J. Clarke, S. D., Ferrell, A., & Karon, S. L.  (2019). *Non-Fatal Opioid Overdose and Associated Health Outcomes: Final Summary Report*. U.S. Department of Health and Human Services. https://aspe.hhs.gov/sites/default/files/migrated_legacy_files//190846/Nonfatal.pdf
